# Mucosal and blood-brain barrier transport kinetics of the plant *N*-alkylamide spilanthol using in vitro and in vivo models

**DOI:** 10.1186/s12906-016-1159-0

**Published:** 2016-06-13

**Authors:** Lieselotte Veryser, Lien Taevernier, Tanmayee Joshi, Pratima Tatke, Evelien Wynendaele, Nathalie Bracke, Sofie Stalmans, Kathelijne Peremans, Christian Burvenich, Martijn Risseeuw, Bart De Spiegeleer

**Affiliations:** Drug Quality and Registration (DruQuaR) group, Faculty of Pharmaceutical Sciences, Ghent University, Ottergemsesteenweg 460, B-9000 Ghent, Belgium; C.U. Shah College of Pharmacy, S.N.D.T. Women’s University, Santacruz (W), Mumbai, 400 049 India; Department of Medical Imaging and Small Animal Orthopedics, Faculty of Veterinary Medicine, Ghent University, Salisburylaan 133, B-9820 Merelbeke, Belgium; Department of Comparative Physiology and Biometrics, Faculty of Veterinary Medicine, Ghent University, Salisburylaan 133, B-9820 Merelbeke, Belgium; Laboratory of Medicinal Chemistry, Faculty of Pharmaceutical Sciences, Ghent University, Ottergemsesteenweg 460, B-9000 Ghent, Belgium

**Keywords:** Plant *N*-alkylamide spilanthol, Caco-2 cells, Oral absorption, Blood-brain barrier

## Abstract

**Background:**

*N*-alkylamides (NAAs) are a large group of secondary metabolites occurring in more than 25 plant families which are often used in traditional medicine. A prominent active NAA is spilanthol. The general goal was to quantitatively investigate the gut mucosa and blood-brain barrier (BBB) permeability pharmacokinetic properties of spilanthol.

**Methods:**

*Spilanthes acmella* (L.) L. extracts, as well as purified spilanthol were used to investigate (1) the permeation of spilanthol through a Caco-2 cell monolayer in vitro, (2) the absorption from the intestinal lumen after oral administration to rats, and (3) the permeation through the BBB in mice after intravenous injection. Quantification of spilanthol was performed using a validated bio-analytical UPLC-MS^2^ method.

**Results:**

Spilanthol was able to cross the Caco-2 cell monolayer in vitro from the apical-to-basolateral side and from the basolateral-to-apical side with apparent permeability coefficients P_app_ between 5.2 · 10^−5^ and 10.2 · 10^−5^ cm/h. This in vitro permeability was confirmed by the in vivo intestinal absorption in rats after oral administration, where an elimination rate constant k_e_ of 0.6 h^−1^ was obtained. Furthermore, once present in the systemic circulation, spilanthol rapidly penetrated the blood-brain barrier: a highly significant influx of spilanthol into the brains was observed with a unidirectional influx rate constant K_1_ of 796 μl/(g · min).

**Conclusions:**

Spilanthol shows a high intestinal absorption from the gut into the systemic circulation, as well as a high BBB permeation rate from the blood into the brain.

**Electronic supplementary material:**

The online version of this article (doi:10.1186/s12906-016-1159-0) contains supplementary material, which is available to authorized users.

## Background

*N*-alkylamides (NAAs) are a large group of secondary metabolites occurring in more than 25 plant families, often used in traditional medicine and claimed to possess a diverse range of pharmacological activities such as antimicrobial, analgesic and anti-inflammatory properties [1–8]. The NAAs consist of a short-chain amine linked to an aliphatic chain of poly-unsaturated fatty acids through a central peptide amide bond. Spilanthol (affinin; deca-2E,6Z,8E-trienoic acid isobutylamide; F3M1 according to the FxMy classification of NAAs) is a highly abundant and biologically potent triene NAA found in Asteracea plants such as *Spilanthes acmella*. Traditionally, *Spilanthes acmella* plants are not only used as a food spice, in toothpastes and cosmetics, but have also been used in folk medicine for the treatment of toothaches, stomatitis, rheumatism, fever, funal skin infections and diverse pain and neuropathic disorders [8]. The pharmacological central nervous system (CNS) activity and physiological mechanisms of spilanthol are only fragmentary documented. Chakraborty et al. [9] and Barman et al. [10] reported a central analgesic activity of a subcutaneously *Spilanthes acmella* extract in an in vivo rat tail flick experiment. Rios et al. [11] showed that a *Heliopsis longipes* extract, in which spilanthol was the main active compound, evoked cortical GABA (gamma-aminobutyric acid) release in an ex vivo in vitro mice brain-tissue study.

In order to exert these CNS effects, these compounds must be able to cross several physiological barriers in the human body. Using in vitro Franz diffusion cell (FDC) experiments, it has already been shown that the *N-*alkylamide spilanthol can permeate the human skin and pig oral mucosa, reaching the systemic circulation after topical application [12–14]. The permeability coefficient K_p,aq_ was 3.31 · 10^−3^ cm/h for human skin and 11.3 · 10^−3^ cm/h for the porcine buccal mucosa.

After oral administration, compounds must be able to pass through the intestinal barrier to reach the systemic circulation. Matthias et al. [15] investigated the transport of mainly diene *N*-alkylamides from *Echinacea* (*E. angustifolia* and *E. purpurea* root) through a Caco-2 cell monolayer, which is a model of the intestinal barrier, and found that after 90 min, more than 50 % of the NAAs permeated through the monolayer.

Once present in the blood by passsage through the skin or mucosa, compounds can cross the blood-brain barrier to exert central nervous system effects. This entry of compounds into the brain is strictly regulated and controlled: the BBB forms not only a physical barrier (tight junctions), but also a transport and metabolic barrier (enzymes) to maintain an adequate microenvironment of the neuronal cells [16]. A previous study indicated that tetraene NAAs from *Echinacea* appeared in the brains after a single oral dose of 2.5 mg/kg to rats [17].

Up till now, there is no information about the transport kinetics of the 2,6,8-triene *N*-alkylamide spilanthol through the intestinal barrier after oral application and through the blood-brain barrier, once present in the blood. Therefore, the aim of this study was to quantitatively investigate the intestinal permeability (in vitro Caco-2 cell monolayer and in vivo oral gavage rat model) and the BBB transport kinetics (in vivo mice model) of spilanthol. Moreover, the distribution of spilanthol within the BBB, *i.e.* brain parenchyma versus endothelial cells, was investigated.

## Methods

### Chemicals and reagents

Ultrapure water (H_2_O) of 18.2 MΩ.cm quality was produced by an Arium 611 purification system (Sartorius, Göttingen, Germany). Vitamin E-TPGS, Hanks’ Balanced Salt Solution (HBSS), trypan blue, potassium chloride (KCl), dimethylsulfoxide (DMSO), phosphate buffered saline (PBS), sodium chloride (NaCl), calcium chloride dehydrate (CaCl_2_.2H_2_O), sodium lactate, dichloromethane, sodium hydrogen carbonate (NaHCO_3_), sodium sulphate (Na_2_SO_4_), sodium dihydrogen phosphate (NaH_2_PO_4_), hydrochloric acid (HCl) and urethane were purchased from Sigma-Aldrich (Diegem, Belgium), while bovine serum albumin (BSA), disodium hydrogen phosphate dehydrate (Na_2_HPO_4_.2H_2_O), sodium dihydrogen phosphate monohydrate (NaH_2_PO_4_.H_2_O), sodium hydrogen carbonate and absolute ethanol (≥99.9 % V/V) were obtained from Merck KGaA (Darmstadt, Germany). Absolute ethanol (99.8 % V/V), HPLC gradient grade methanol (MeOH) and acetonitrile (ACN) came from Fisher Scientific (Erembodegem, Belgium). Dextran was obtained from AppliChem GmbH (Darmstadt, Germany). UPLC-MS grade MeOH and ACN were bought from Biosolve (Valkenswaard, the Netherlands). Phosphoric acid (85 %) (H_3_PO_4_) and dimethylacetamide were purchased from Jansen Chimica (Geel, Belgium). Trypsin-EDTA was obtained from Invitrogen (Ghent, Belgium). Calcium dichloride (CaCl_2_), LC-MS grade formic acid (FA), polyethylene glycol 400 (PEG 400), tween 80, D-glucose, sodium hydroxide (NaOH) and HEPES were purchased at Fluka (Diegem, Belgium) while propylene glycol (PG) was bought from Riedel-de Haën (Seelze-Hannover, Germany). Triethylamine, decanoyl chloride and isobutylamine were purchased at Acros Organics (Geel, Belgium). The BBB-positive control dermorphin was purchased at Bachem (Bubendorf, Switzerland). NMR solvents were bought from Eurisotop (Saint-Aubin, France).

### Products examined

The ethanolic *Spilanthes acmella* (L.) L. flowers extract (batch 0001853516, 30 % w/w spilanthol in ethanol) was obtained from Robertet (Grasse, France) and was used for the BBB transport assay. *Spilanthes acmella* (L.) L. is also known as Akarkara (local name) and Para Cress (English name). HPLC analysis confirmed the spilanthol content, as well as a small quantity (9 %) of other NAAs [14]. The *Spilanthes acmella* (L.) L. extract (A. Vogel, label claim indicated 2 % w/w dry residue of extracts in 69 % V/V ethanol; internal assay: 0.11 % w/w spilanthol, purity >90 %) came from Bioforce AG (Switzerland) and was used for the oral gavage experiment.

Purified spilanthol was used for the Caco-2 cell permeability assay. Semi-preparative HPLC was used for its purification as follows: the *Spilanthes acmella* (L.) L. extract of Robertet was dissolved in 30:70 (V/V) H_2_O:ethanol and filtered using a 0.45 μm nylon HPLC filter (Whatman). One ml of this solution was injected on a Vydac C18 monomeric semi-preparative column (Grace, 250 mm x 10 mm, 5 μm particle size, 300 Å pore size) using a Waters HPLC, equipped with a Waters 2487 Dual Absorbance Detector. The sample compartment and column temperature were maintained at room temperature. An isocratic elution mode was used with a 50:50 (V/V) A:B (A: 0.1 % FA in H_2_O and B: 0.1 % FA in MeOH) mobile phase. A flow rate of 6.0 ml/min was used and UV detection was performed at 237 nm. Fractions between 16 and 32 min retention time were collected and lyophilized using a Christ Gamma 1–16 LSC freeze-dryer (Q-lab, Vilvoorde, Belgium). The purity of the isolated spilanthol was determined by analytical HPLC-UV at 237 nm [12] and was 99.9 %.

**Fig. 1:**
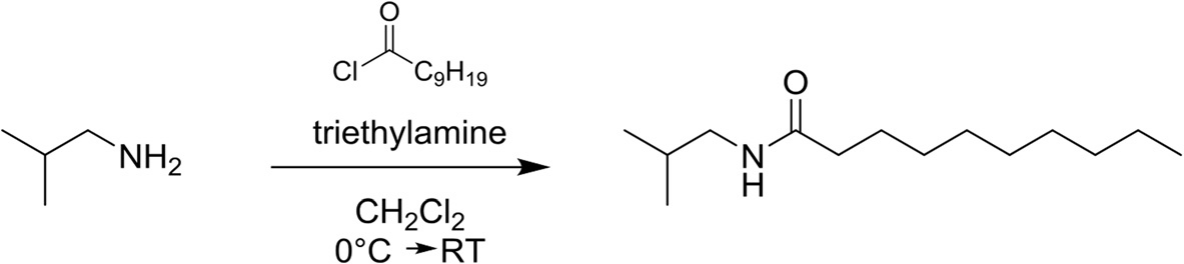
Synthesis of isobutyldecanamide (analytical internal standard)

As analytical internal standard (IS) in the bio-analytics, isobutyldecanamide was used and synthesised as described in Fig. 1. The NMR spectra of this internal standard can be retrieved in Additional file 1.

Triethylamine (6.0 mmol, 608 mg, 837 μl) was added to a solution of isobutylamine (6.0 mmol, 439 mg, 597 μl) in dichloromethane (30 ml). The mixture was cooled to 0 °C using an ice bath. Decanoyl chloride (5.0 mmol, 954 mg, 1.0 ml) was slowly added to the solution. The mixture was stirred overnight, allowing the temperature to rise to room temperature. The reaction mixture was extracted with 50 ml dichloromethane which was washed successively with HCl (1.0 M aq.) and NaHCO_3_ (sat. aq.). The organic layer was dried on Na_2_SO_4_, filtered and concentrated *in vacuo* to yield the title compound (1.07 g, 4.7 mmol) as an off-white waxy solid. The compound was identified by ^1^H and ^13^C NMR recorded with a Varian Mercury-300BB (300/75 MHz) spectrometer. Chemical shifts are given relative to tetramethylsilane (0 ppm) ^1^H NMR (300 MHz, CDCl_3_): δ 5.47 (s, 1H), 3.07 (t, *J* = 6.45 Hz, 2H), 2.16 (t, *J* = 7.44 Hz, 2H), 1.80–1.69 (m, 1H), 1.68–1.58 (m, 2H) 1.38–1.18 (m, 12H), 0.90 (d, *J* = 6.90 Hz, 6H), 0.87 (t, J = 6.90 Hz, 3H); ^13^C NMR (75 MHz, CDCl_3_): δ 173.25, 46.92, 37.16, 32.00, 29.61, 29.51, 29.47, 29.41, 28.67, 26.05, 22.80, 20.24, 14.24.

### In vitro permeation study in Caco-2 cell monolayers

#### Cell culture

Caco-2 cells, originating from a human colorectal carcinoma were maintained in Dulbecco’s modified Eagle’s medium (DMEM) at 95 % humidity and 37 °C in an atmosphere of 5 % CO_2_, supplemented with 10 % (V/V) fetal bovine serum, 2 mM L-glutamine and 1 % non-essential amino acids (100x), 100 U/ml penicillin and 100 μg/ml streptomycin (all from Invitrogen/GIBCO, Ghent, Belgium).

#### Caco-2 cell permeability assay

The Caco-2 cell intestinal model was used as a model for the gut mucosa to investigate the permeation of spilanthol. Caco-2 cells were seeded at a density of 2.6 · 10^5^ cells/cm^2^ cells for each Transwell (Corning Costar, New York, USA) membrane insert filter (0.4 μm pore size, 12 mm filter diameter) and cultivated in the described supplemented DMEM, also containing 100 units/ml penicillin and 100 μg/ml streptomycin. The medium was changed every second day. The cells were allowed to grow and differentiate for 21–29 days until monolayers were formed. Intact membranes/integrity of the monolayers were checked via the measurement of transepithelial electrical resistance (TEER) of the monolayers using the Millicell-ERS system (Millipore Corp., Bedford, MA, USA) before and after transport experiments. The experiment was performed in duplicate for each dose solution. Transport experiments were carried out in the apical-to-basolateral (ab) direction and in the basolateral-to-apical (ba) direction in Hanks’ Balanced Salt solution, according to Hubatsch et al. [18]. Two dose solutions of purified spilanthol at 10 μg/ml were tested, differing in solvent: 0.5 % ethanol (further indicated as DS1) and 0.5 % of a mixture of vitamin E-TPGS (33.3 %), ethanol (11.1 %), PEG 400 (33.3 %) and PG (22.2 %) (further indicated as DS2) dissolved in HBSS.

Final volumes of 0.4 ml apically and 1.2 ml basolaterally were used for 12-mm filter supports during the transport experiment. Samples were taken at 15, 30, 60, 90 and 120 min from the acceptor (= receiver) compartment (basolateral to apical transport: 100 μl; apical to basolateral transport: 300 μl) and were immediately replaced by fresh HBSS. At the last time point, a sample from the donor compartment was taken as well, allowing to obtain the mass balance, which for spilanthol ranged between 104.7 and 120.9 % total recovery. Atenolol (50 μM) and propranolol (20 μM) were used as the low- and high-permeability control, respectively, confirming the validity of the test [19]. Quantification of spilanthol was conducted using the UPLC-MS^2^ method as described below.

The apparent permeability coefficient (P_app_ in cm/s) of spilanthol was calculated from a non-sink equation. From Fick’s first law: J = − D × [dC(x)]/dx (D is the diffusion coefficient, J is the flux or transfer rate along the donor-to-receptor side, x is the distance from the donor compartment and C(x) is the concentration in the barrier at the coordinate x in the barrier), the following differential equation is derived: dM_r_(t)/dt = P_app_ × A × [C_d_(t) − C_r_(t)], in which M_r_ is the amount of substance in the receiver chamber, P_app_ is the apparent permeability coefficient (a product of distribution coefficient with diffusion coefficient divided by the barrier thickness), A is the cross-sectional area of the barrier, C_d_ is the donor concentration and C_r_ is the receiver concentration. This differential equation is the basis to calculate P_app_ values of compounds requiring sink, as well as non-sink conditions with different initial conditions to solve this equation. In the case of non-sink conditions, this resulted in the following solution [18, 20]:

$$ {\mathrm{C}}_{\mathrm{r}}\left(\mathrm{t}\right)=\left[\frac{\mathrm{M}}{{\mathrm{V}}_{\mathrm{d}}+{\mathrm{V}}_{\mathrm{r}}}\right]+\left({\mathrm{C}}_{\mathrm{r},\mathrm{t}-1}\times \mathrm{f}-\left[\frac{\mathrm{M}}{{\mathrm{V}}_{\mathrm{d}}+{\mathrm{V}}_{\mathrm{r}}}\right]\right)\times {\mathrm{e}}^{-{\mathrm{P}}_{\mathrm{app}}\times \mathrm{A}\times \left(\frac{1}{{\mathrm{V}}_{\mathrm{d}}}+\frac{1}{{\mathrm{V}}_{\mathrm{r}}}\right)\times \Delta \mathrm{t}} $$

in which t is the time (s), V_d_ is the volume of the donor compartment (ml), V_r_ the volume of the receiver compartment (ml), A the area of the filter (= 1.12 cm^2^), M the total amount of spilanthol in the system at time t (μg), C_r,t-1_ the concentration of spilanthol in the receiver compartment at the previous time point (μg/ml), f is the sample replacement dilution factor (1-V_s_/V_r_ with V_s_ is the sample volume), Δt is the time at time t minus the previous time point (s) and C_r_(t) the concentration of spilanthol in the receiver compartment at time t (μg/ml). Non-linear curve fitting by minimisation of the sum of squared residuals (SSR) was used to obtain the P_app_. The efflux ratio is calculated as P_app,ba_/P_app,ab_, while the uptake ratio is calculated as P_app,ab_/P_app,ba_.

### In vivo pharmacokinetic experiment with rats

#### Animals

The Wistar rats were bred in Bharat Serum and Vaccines pvt. Ltd, Thane, India (registration number 103/99/CPCSEA) and kept for the experiments at C. U. Shah College of Pharmacy, SNDT Women’s University, Santacruz, Mumbai, India (registration number 39/99/CPCSEA). The half of the rats used in the experiment were female rats and the other half male rats. Rats of 7-8 weeks old, weighing 220 g, and fasted overnight were used for the experiment.

#### Pharmacokinetic experiment

The pharmacokinetic oral gavage experiment was carried out at C. U. Shah College of Pharmacy, SNDT Women’s University, Santacruz, Mumbai, India. A spilanthol dose solution was prepared with the *Spilanthes acmella* (L.) L. extract (A.Vogel). 1.5 ml of a spilanthol dose solution of 0.73 mg spilanthol/g dose solution in 10:20:30:40 (w/w/w/w) ethanol:PG:vitamin E-TPGS:PEG400 was administered to the rats using a gavage needle made up of stainless steel (length 3 inches and 2.5 mm internal diameter). A blank dose solution was used as well, containing ethanol, PG, vitamin E-TPGS and PEG without spilanthol. For each dose solution, six rats were used of which three were females and three males. Blood was collected (1.5 ml) from the retro orbital vein at specified time points *i.e.* 1, 2, 3, 4, 6, and 8 h after oral administration of the dose solution. The collected blood samples were centrifuged using a Remi R-24 centrifuge. At the end of the study, the animals were sacrificed by CO_2_ inhalation. All samples were immediately frozen at -80 °C until bio-analysis. The elimination rate constant was calculated using a one compartmental model using GraphPad software (La Jolla, USA), using the following equation: $\mathrm{C}\left(\mathrm{t}\right)={\mathrm{C}}_0\times {\mathrm{e}}^{-{\mathrm{k}}_{\mathrm{e}}\times \mathrm{t}}$, in which t is time (h), C_0_ the concentration of spilanthol at time t = 0 (ng/ml), C(t) the concentration of spilanthol at time t and k_e_ the elimination rate constant (h^−1^). The elimination half-life (h) is computed as ln(2)/k_e_.

### In vivo blood-brain barrier experiment with mice

#### Animals

Female, Institute for Cancer Research, Caesarean Derived-1 (ICR-CD-1) mice (Harlan Laboratories, Venray, Netherlands) of age 7–10 weeks and weighing 29–32 g, were used during the BBB transport experiments. All animal experiments were performed in accordance with the Ethical Committee principles of laboratory animal welfare as approved by our institute (Ghent University, Faculty of Veterinary Medicine, no. EC2012/157 and no. EC2014/128).

#### Blood-to-brain transport

An in vivo multiple time regression (MTR) analysis was performed to investigate if spilanthol was able to cross the BBB. A dose solution of spilanthol was prepared using the *Spilanthes acmella* (L.) L. extract (Robertet) with a final concentration of 4 mg/ml spilanthol dissolved in 7.0 % ethanol, 2.5 % dimethylacetamide, 0.6 % tween 80 (all w/w) diluted in lactated Ringer’s solution containing 1 % BSA. The ICR-CD-1 mice were anesthetized by intraperitoneal injection with a 40 % (w/V) urethane solution (3 g/kg) and the jugular internalis vein and carotid artery were isolated, 20 μl of the spilanthol dose solution was injected into the jugular vein. Blood was obtained from the carotid artery at regular time points after injection (1, 3, 5, 10, 12.5 and 15 min, with start and end in duplicate), and the mice were immediately thereafter decapitated. After sacrificing the mice, the brains were collected. The collected blood from the carotid artery was centrifuged at 10 000 *g* for 15 min at 21 °C, resulting in serum. In order to assure the validity of the MTR method, ^125^I-BSA and dermorphin diluted in lactated Ringer’s solution containing 1 % BSA were used as the negative and positive control, respectively [21–23]. Furthermore, to evaluate the functional BBB integrity under our experimental conditions, ^125^I-BSA was also administered to the mice in a solution containing the same surfactant and co-solvents in equal concentrations as in the spilanthol dose solution. The serum profile of spilanthol was plotted against the time (expressed in min). The curve was fitted using the same one compartmental model as described in the pharmacokinetic experimental section. In order to determine the BBB permeability of a compound, the ratio of the brain and serum concentration (μl/g) was plotted versus a derived time variable, *i.e.* the exposure time (θ) of the Gjedde-Patlak plot [24, 25].

The exposure time is defined as the integral of the concentration of spilanthol in serum from start (t = 0 min) to time T, divided by the concentration of spilanthol in serum at time T: $\uptheta ={\displaystyle {\int}_0^{\mathrm{T}}\frac{{\mathrm{C}}_{\mathrm{S}}\left(\mathrm{t}\right)\cdot \mathrm{d}\mathrm{t}}{{\mathrm{C}}_{\mathrm{S}}\left(\mathrm{T}\right)}}$. The integral of the concentration of spilanthol in serum from zero to time T is the area under the curve until time T.

A biphasic model of blood-brain transfer was used to fit the uptake, as elaborated by Wong et al. [26]:

$$ \frac{{\mathrm{C}}_{\mathrm{brain}}\left(\mathrm{T}\right)}{{\mathrm{C}}_{\mathrm{S}}\left(\mathrm{T}\right)}=\mathrm{K}\times \uptheta +{\mathrm{V}}_{\mathrm{g}}\times \left(1-{\mathrm{e}}^{\left(-\uptheta \times \left(\frac{{\mathrm{K}}_{1-\mathrm{K}}}{{\mathrm{V}}_{\mathrm{g}}}\right)\right)}\right)+{\mathrm{V}}_0\begin{array}{c}\hfill \mathrm{K}=0\hfill \\ {}\hfill \cong \hfill \\ {}\hfill \hfill \end{array}\ {\mathrm{V}}_{\mathrm{g}}\times \left(1-{\mathrm{e}}^{\left(-\uptheta \times \left(\frac{{\mathrm{K}}_1}{{\mathrm{V}}_{\mathrm{g}}}\right)\right)}\right)+{\mathrm{V}}_0 $$

where K_1_ is the unidirectional clearance (μl/(g · min)), K is the net clearance (μl/(g · min)), V_g_ the tissue brain distribution volume (μl/g), and V_0_ the vascular brain distribution volume (μl/g). The negative control BSA has a low vascular distribution volume and this value (14.8 μl/g) is used as V_0_ to calculate the brain kinetic parameters of spilanthol. In the equation, C_brain_(T) is the concentration of spilanthol in the brain at time T (ng/g) and C_s_(T) the concentration of spilanthol in serum at time T (ng/μl).

#### Capillary depletion

To investigate the distribution of spilanthol in the parenchyma and capillaries of the brain, a capillary depletion experiment was performed. By means of this experiment, it is possible to distinguish the transport of spilanthol into the brain (represented by the parenchyma) and part of spilanthol which is trapped by the endothelial cells of the brain (represented by the capillaries). The method of Triguero et al. [27], as modified by Gutierrez et al. [28], was used [29]. Briefly, after anesthetizing the mice intraperitoneally with 40 % (w/V) urethane solution (3 g/kg), 20 μl of the 4 mg/ml spilanthol dose solution was injected into the jugular vein. Blood was collected from the abdominal aorta ten min after injection and serum was obtained by centrifuging the blood at 10 000 *g* during 15 min at 21 °C. Immediately after blood collection, the skin of the mice’s chest is removed to clamp the aorta and the jugular veins are severed. Immediately after clamping the aorta, the brain is perfused manually with 20 ml of Lactated Ringer’s solution. The mice are decapitated immediately after perfusion and brain is collected. The brains were put into an Eppendorf tube and weighed. 525 μl ice-cold capillary buffer (10 mM HEPES, 141 mM NaCl, 4 mM KCl, 2.8 mM CaCl_2_, 1 mM MgSO_4_, 1 mM NaH_2_PO_4_ and 10 mM D-glucose adjusted to pH 7.4) was added and homogenized. Then, 1000 μl of 26 % ice-cold dextrane solution in capillary buffer was added and vortexed. The Eppendorf tube was centrifuged at 20 000 *g* for 60 min at 4 °C. Pellet (capillaries) and supernatant (parenchyma and fat tissues) were separately collected into an Eppendorf tube and weighed. The sample preparation of the pellet is the same sample preparation method as used for the mice brains, while the sample preparation for the supernatant is the same sample preparation method as used for mice serum, as described in the bio-analytical section below.

The distribution was calculated as follows:

$$ \mathrm{Fraction}\ \left(\%\right)=\frac{{\mathrm{M}}_{\mathrm{tissue}}}{{\mathrm{M}}_{\mathrm{capillaries}}+{\mathrm{M}}_{\mathrm{parenchyma}}}\times 100 $$

where M_tissue_ represents the amount of spilanthol in the capillaries, respectively parenchyma; M_capillaries_ the amount of spilanthol in the capillaries and M_parenchyma_ the amount of spilanthol in the parenchyma.

#### Brain-to-blood transport

To evaluate the efflux of spilanthol out of the brain, an in vivo method previously described was used [29]. Briefly, after anesthetizing the ICR-CD-1 mice using a 40 % (w/V) urethane solution (3 g/kg), the skin of the skull was removed. Thereafter, a hole was made into the lateral ventricle using a 22 G needle marked with tape at 2 mm at the following coordinates: 1 mm lateral and 0.34 mm posterior to the bregma. 1 μl of the 4 mg/ml spilanthol dose solution as used for the blood-to-brain influx experiment was injected intracerebroventriculary (ICV) using a syringe pump (KDS100, KR analytical, Cheshire, UK) at a speed of 360 μl/h for 10 s. At specified time points post-injection (1, 3, 5, 10, 12.5 and 15 min), mice were decapitated. Prior to decapitation, blood was collected from the abdominal aorta. Serum was obtained by centrifuging the blood at 10 000 *g* during 15 min at 21 °C and brains were collected. The efflux brain half-life (t_1/2,brain_) (min) was calculated from the linear regression of the natural logarithm of the spilanthol concentration in brain (ng/g) versus time as follows: t_1/2,brain_ = ln(2)/k_out_, where k_out_ (min^−1^) is defined as the efflux rate constant calculated as the negative value of the slope of the linear regression, applying first order kinetics.

### Bio-analytics

#### Serum sample preparation

60 μl of the serum samples, 60 μl of 4 % (V/V) aqueous H_3_PO_4_ solution and 30 μl of the IS solution were transferred into a 0.5 ml (LoBind Eppendorf) tube and vortexed. Interfering compounds were removed by solid phase extraction using a positive pressure-96 processor (Waters). 100 μl (rat serum) or 125 μl (mice serum) of the previous sample solution was loaded on the HLB Oasis® μelution 96 well plate (Waters, Zellik, Belgium), which was preconditioned with 200 μl of MeOH and equilibrated using 200 μl of ultrapure water. After the loading step, the HLB Oasis® μelution plate was washed using 200 μl of 5 % MeOH in H_2_O followed by 200 μl of 20 % MeOH in H_2_O. Spilanthol was eluted using two times 25 μl ACN. To the eluate, 25 μl of a 80:20 (V/V) H_2_O:MeOH solution was added and analysed with a UPLC-MS^2^ method, as further described.

A basic validation of the bio-analytical method for the quantification of spilanthol in serum was performed, based upon the EMA guideline on bio-analytical method validation (EMEA/CHMP/EWP/192217/2009) [30]. The limit of detection (LoD) (S/N = 3) and limit of quantification (LoQ) (S/N = 10) of spilanthol, determined on the reference standard, were calculated as 0.09 ng/ml and 0.31 ng/ml, respectively, which correspond to 0.17 ng/ml and 0.58 ng/ml in rat serum, respectively. The LoD and LoQ of spilanthol in mouse serum were 0.048 ng/ml and 0.16 ng/ml, respectively. A matrix factor of 1.1 was observed for spilanthol in rat and mouse serum. The quantification of spilanthol in the rat serum samples was performed using a matrix spiked calibration curve (R^2^ = 0.9998). Linearity was assured in a working range from 0.31 ng/ml up to 101 ng/ml, corresponding to 0.58 ng/ml to 189 ng/ml in rat serum. The quantification of spilanthol in the mouse serum samples was performed using a one-point calibration curve with a spilanthol standard in 40:33.33:26.67 (V/V/V) MeOH:ACN:H_2_O. The accuracy of the references used for the calibration curves with the pre-extracted spiked matrix samples and the QC reference pre-extracted spiked matrix samples conformed to the specification limits (< 15 % of the nominal value and for the lower limit of quantification (LLoQ) < 20 % of the nominal value), except for the LLoQ used for the validation of spilanthol in mouse serum (> 20 % of the nominal value). These values are slightly higher than the specification limits given by the EMA in their guidelines for formal bio-analytical method validation, but are still acceptable for our purposes in the discovery phase. Precision was expressed as the coefficient of variation (CV). The within-run CV value did not exceed 15 % for the pre-extracted spiked matrix samples. Also the LLoQ did not exceed 20 % and are thus all conform to the specification limits. The recovery in rat serum was 102.1 % (18.8 ng/ml to 189 ng/ml in serum concentration range), calculated from the slopes of the pre-extracted and post-extracted spiked matrix samples. The recovery in mouse serum was 113.3 % (3 to 257 ng/ml in serum concentration range). No significant carry-over was observed (< 20 % of LLoQ spilanthol and < 5 % for IS). The selectivity was conform to the specification limits (placebo rat serum < 20 % of LLoQ spilanthol and < 5 % for IS).

#### Brain sample preparation

For the analysis of the mice brain samples, the weighed brains were transferred into a test tube and crushed. 1.0 ml of the IS solution in ACN was added, followed by shaking during 4 h at 110 rpm at room temperature using an Eppendorf centrifuge 5810 (Eppendorf, Rotselaar, Belgium). The test tube was centrifuged at 250 *g* for 5 min and 800 μl of the supernatant was transferred into an Eppendorf tube. Thereafter, a second centrifugation was performed at 20 000 *g* during 5 min at room temperature. 750 μl of the supernatant was evaporated to dryness under nitrogen and 175 μl 90:10 (V/V) H_2_O:ACN was added to redissolve spilanthol. Thereafter, 160 μl of this solution was loaded on the HLB Oasis® μelution 96 well plate. The same washing and elution steps were used as for the serum samples.

Again, a basic validation of the bio-analytical method for the quantification of spilanthol in brains was performed, based upon the EMA guideline on bio-analytical method validation (EMEA/CHMP/EWP/192217/2009) [29]. The LoD and LoQ of spilanthol were 6.30 pg/g in brain and 21.0 pg/g in brain, respectively. Ion enhancement (matrix factor = 1.38 > 1) was observed for the quantification of spilanthol in mice brains. Hence, quantification of spilanthol in the brain samples was performed using the matrix spiked calibration curve (R^2^ = 0.9986). Linearity was assured in a working range of 0.107 ng/ml to 175 ng/ml spilanthol, corresponding to 21.0 pg/g to 34.3 ng/g in brain. The accuracy of the references used for the calibration curves with the pre-extracted spiked matrix samples conformed to the specification limits (< 15 % of the nominal value), except for the LLoQ used for the validation of spilanthol in brains (> 20 % of the nominal value), but was still acceptable for our purposes in the discovery phase. The within-run CV value did not exceed 15 % for the pre-extracted spiked samples. Also the LLoQ did not exceed 20 % and are thus all conform to the limits. The recovery in mouse brains was 72.7 % (0.92 to 98.10 ng/g brain concentration range).

#### UPLC-MS^2^ method

Quantification of spilanthol was performed using a UPLC-MS^2^ method, which was developed on an Acquity UPLC coupled to a Xevo™ TQ-S mass spectrometer (MS) (Waters, Zellik, Belgium) with electrospray ionisation source and a triple quadrupole mass analyser. An Acquity UPLC RP C18 column (Waters, 50 x 2.1 mm, 1.7 μm) with a suitable guard column was used. The sample compartment was kept constant at 5 °C, while the column temperature was maintained at 30 °C. 2 μl of the sample was injected and the flow rate was set to 0.5 ml/min. A mobile phase was applied using solvent A (0.1 % FA in 30:70 (V/V) H_2_O:MeOH) and solvent B (0.1 % FA in MeOH) in gradient mode as follows: 0–1.6 min 100:0 (V/V) A:B*,* 1.6–2 min going from 100:0 (V/V) A:B to 0:100 (V/V) A:B, 2–3 min 0:100 (V/V) A:B, 3–3.4 min going from 0:100 (V/V) A:B to 100:0 (V/V) A:B, 3.4–5 min 100:0 (V/V) A:B. The needle wash solvent was 60:40 (V/V) DMSO:ACN. The MS was operated in the positive electrospray ionisation mode (ESI^+^), with an optimised capillary voltage of 3.0 kV, cone voltage of 50 V and source offset of 60 V. Source and desolvation temperatures were set at 150 °C and 500 °C, respectively, while cone and desolvation gas (N_2_) flows were 180 and 1000 l/h, respectively. Acquisition was performed in the multiple reaction monitoring (MRM) mode with *m/z* 222.15 to *m/z* 80.96 transition. The applied collision energy was 20 eV (collision gas = argon). Data were acquired and analysed through MassLynx® software (V4.1 SCN 843, Waters).

## Results

### Caco-2 cell permeability

**Fig. 2:**
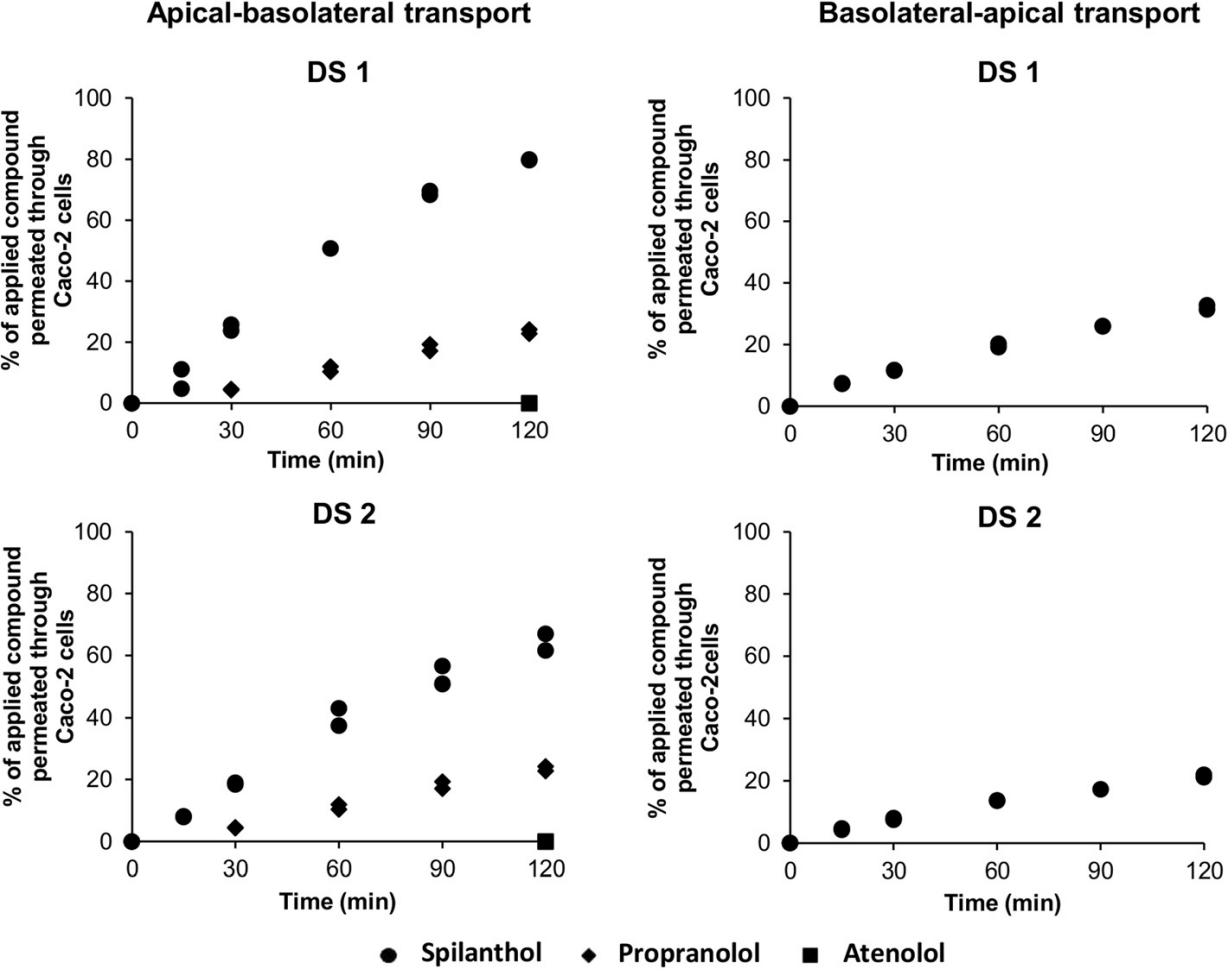
Apical-to-basolateral transport and basolateral-to-apical transport of spilanthol in DS1 and DS2

Propranolol was used as a positive control and a P_app,ab_ value of 19.1 · 10^−6^ cm/s was obtained, which is in good agreement with the values reported in literature [31]*.* Atenolol served as the negative control and a lower permeability was observed compared to propranolol. Spilanthol was able to diffuse through the Caco-2 cell monolayer from the apical-to-basolateral side, as well as from the basolateral-to-apical side. Figure 2 shows the percentages of spilanthol permeated through the Caco-2 cells versus time plot with DS1 and DS2.

The percentage of the applied spilanthol which permeated through the Caco-2 cells from the apical-to-basolateral membrane after 120 min is 79.8 % (or 3.20 μg spilanthol) with DS1 and 64.3 % (or 2.47 μg spilanthol) with DS2. In the opposite direction, the percentage of spilanthol which permeated through the Caco-2 cells from the basolateral-to-apical membrane after 120 min is 32.1 % (or 3.86 μg spilanthol) with DS1 and 21.6 % (or 2.49 μg spilanthol) with DS2.

The apparent permeability coefficient (P_app_) of spilanthol obtained with DS1 and DS2 from the apical-to-basolateral side is 8.42 ± 0.59 · 10^−5^ cm/s (mean ± SD, *n* = 2) and 5.61 ± 0.54 · 10^−5^ cm/s (mean ± SD, *n* = 2), respectively. The P_app_ of spilanthol obtained with DS1 and DS2 was also determined from the basolateral-to-apical side and is 10.21 ± 0.46 · 10^−5^ cm/s (mean ± SD, *n* = 2) and 5.20 ± 0.06 · 10^−5^ cm/s (mean ± SD, *n* = 2), respectively. The efflux ratio for spilanthol with DS1 and DS2 is 1.21 and 0.93, respectively, while the uptake ratio for spilanthol with DS1 and DS2 is 0.82 and 1.08, respectively.

### Oral gavage experiment

**Fig. 3:**
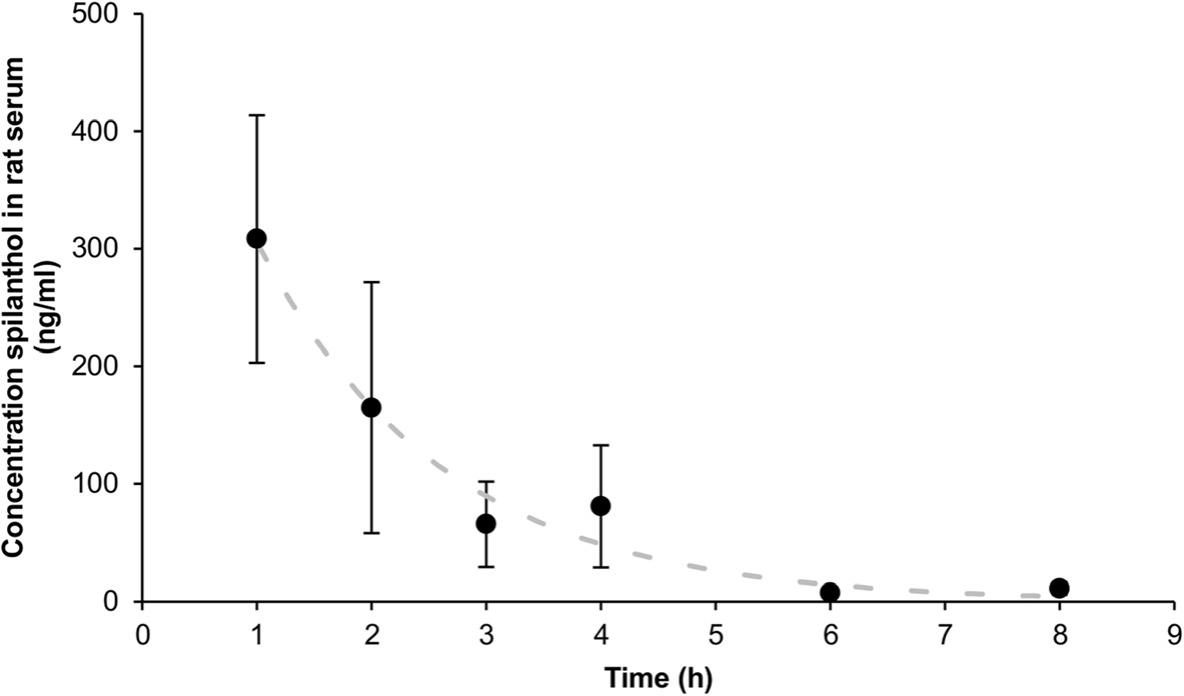
Concentration of spilanthol in rat serum as a function of time after oral gavage, one compartmental model (*n* = 3, mean, error bars: SEM)

Figure 3 represents the rat serum data after oral gavage of spilanthol, fitting a one compartmental model with the mean concentration of spilanthol at each time point. These results of the in vivo experiment confirm the in vitro Caco-2 cell monolayer results of spilanthol.

From these data, the elimination rate constant and the elimination half-life were calculated as 0.61 h^−1^ and 1.13 h, respectively.

### Blood-brain barrier transport kinetics of spilanthol

**Fig. 4:**
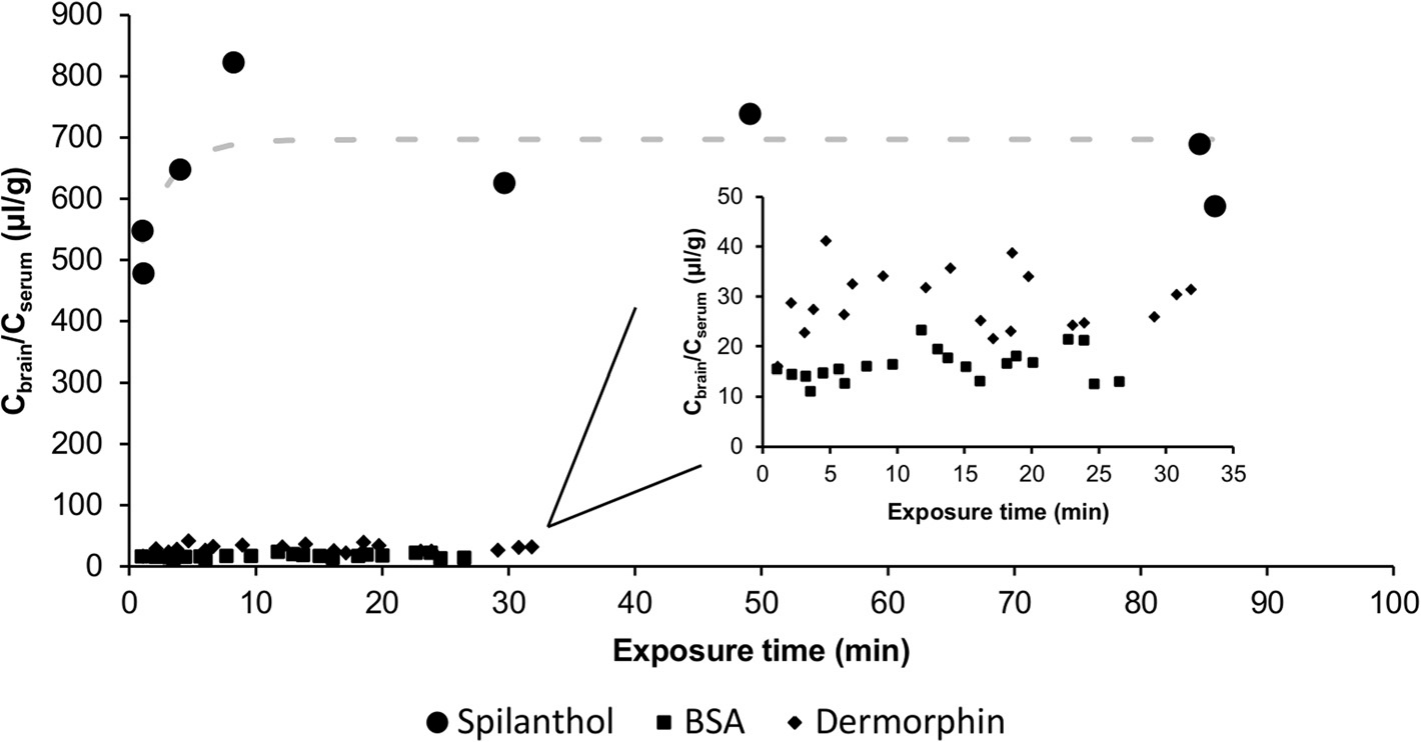
Brain influx results of spilanthol, BSA and dermorphin (MTR in mice)

The blood-to-brain transport of spilanthol was investigated. The negative and the positive control confirmed the validity of the BBB test. The K_1_ value of BSA (negative control) was 0.12 μl/(g · min), while the K_1_ value of the positive control dermorphin was 0.26 μl/(g · min), both consistent with previous data [29, 32]. The multiple time regression data indicated that spilanthol crosses the blood-brain barrier. In Fig. 4, the ratio of the concentration of spilanthol in brain and serum is plotted versus the exposure time. Furthermore, the kinetic influx data of ^125^I-BSA with or without the surfactant/so-solvents were not significant different, indicating that the surfactant and co-solvents did not affect the BBB integrity (data not shown).

The MTR data of spilanthol were fitted using a biphasic model, based on the modified Gjedde-Patlak equation according to Wong et al. [26]. A rapid but highly significant influx of spilanthol into the brains was observed with a unidirectional influx rate (K_1_) of 796 μl/(g · min). The tissue brain distribution volume (V_g_) is 652 μl/g.

The curve reached a plateau-phase after about 10 min exposure time and can be explained by efflux of spilanthol out of the brain.

At the same time, also the elimination kinetics of spilanthol in serum were evaluated in the mice model. The serum profile of spilanthol follows a one compartmental model. The concentration of spilanthol at time zero was 3.05 μg/ml and an elimination rate constant and elimination half-life of 0.22 min^−1^ and 3.16 min were obtained.

### Capillary depletion

The capillary depletion method was used to study the distribution of spilanthol *i.e.* the part that was taken up by the brain and the part which was trapped in the endothelial cells of the brain capillaries. A high brain penetration of spilanthol was found: about 98 % of spilanthol (corresponding to 137.8 μl/g) was found in the brain parenchyma and only about 2 % of spilanthol (corresponding to 2.4 μl/g) that entered the brain remained in the brain capillaries.

### Brain-to-blood transport kinetics of spilanthol

**Fig. 5:**
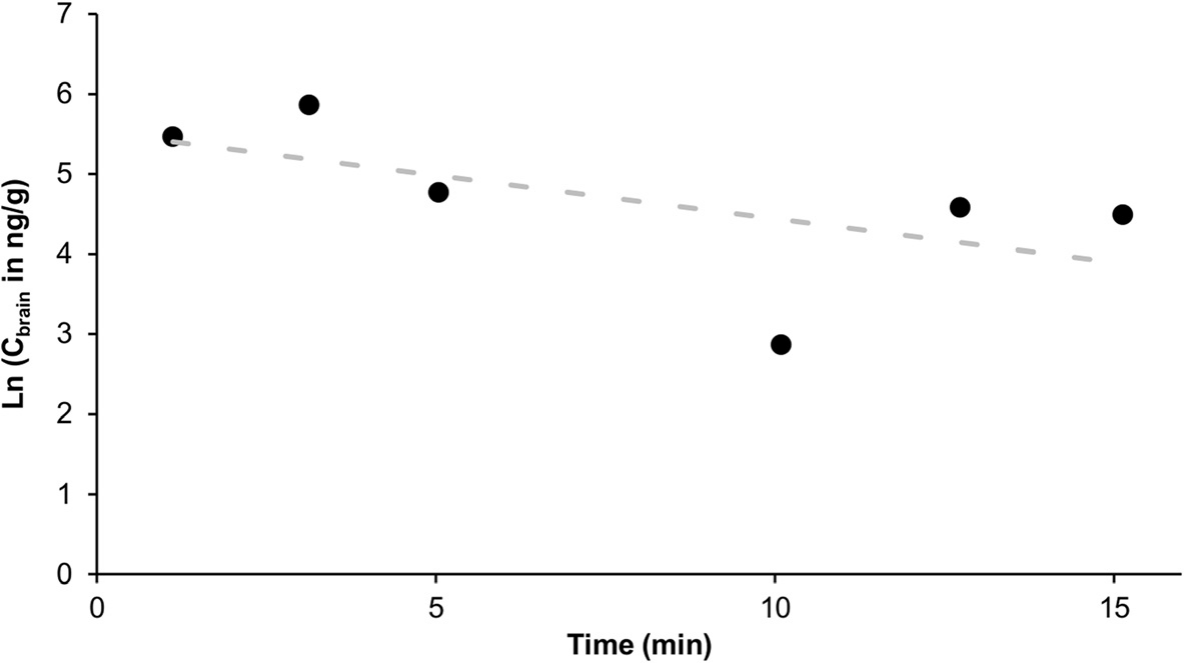
Brain efflux of spilanthol

The efflux properties of spilanthol out of the brain were investigated by quantifying the concentration of spilanthol in the brain after intracerebroventricular injection of the dose solution. When evaluating the transport of spilanthol out of the brain into the blood, it is confirmed that there is also efflux of spilanthol (Fig. 5), which can explain the rapid plateauing observed when evaluating the blood-to-brain kinetics during the influx experiment (Fig. 4). The efflux transfer constant k_out_ was derived from the absolute value of the slope of the natural logarithm of the concentration of spilanthol in the brain (ng/g) versus the experimental time curve (min). The k_out_ calculated for spilanthol was 0.11 min^−1^, equal to a t_1/2,brain_ of 6.4 min.

## Discussion

In this study, the in vitro Caco-2 cell monolayer permeability of spilanthol, a 2,6,8-triene NAA, was investigated using two different dose solutions. The permeation level of spilanthol through Caco-2 cells was the highest from the apical-to-basolateral side: 64–80 % of the applied DS permeated through Caco-2 cells after 120 min (absorptive direction). Spilanthol, applied as DS2, did not appear to better permeate the Caco-2 cells compared to spilanthol applied as DS1, suggesting that co-solvents (vitamin E-TPGS, PEG 400, PG) did not enhance the permeation of spilanthol through the cell monolayer. Both dose solutions did not contain more than 0.5 % ethanol in buffer, as final ethanol concentrations above 2 % can damage the Caco-2 cell monolayer.

As mentioned earlier, Matthias et al. [15] investigated a different type of NAA, namely the transport of 2-ene and 2,4-diene NAAs through the Caco-2 cell monolayer (dissolved in ethanol and diluted in HEPES buffer, final ethanol concentration did not exceed 2 %) with P_app_ values ranging from 3 · 10^−6^ to 3 · 10^−4^ cm/s. The diversity in P_app_ for the different *N*-alkylamides was correlated to structural variations, with saturation and N-terminal methylation contributing to lowered P_app_ values.

The P_app_ values obtained for spilanthol in this study were all above 1 · 10^−6^ cm/s, indicating an almost complete intestinal absorption. Compounds with P_app_ values < 1 · 10^−6^ cm/s show a less readily absorption. The P_app_ values obtained for spilanthol in this study (5 · 10^−5^ to 10 · 10^−5^ cm/s) are thus consistent with the structural conclusions reported by Matthias et al. [15] based on the mono- and diene-NAAs from *Echinacea* plant species.

It has been reported that if there is a more than 2-fold difference in P_app_ values between the apical-to-basolateral and the basolateral-to-apical direction, a high probability of active transport exists [33]. In this experiment, for both dose solutions of spilanthol, this difference was less than 2-fold, indicating that no active transport function can be readily assumed. Studies have proven a correlation between drug permeability coefficients obtained during a Caco-2 cell monolayer experiment and oral absorption in humans [31, 34, 35]. However, as no in vitro model can yet totally mimic the in vivo intestinal barrier, since many factors such as solubility, formulation, chemical composition, pH of the intestinal secretions, food composition, gastric emptying time, intestinal motility and blood flow play a role [33, 36, 37], an oral gavage experiment in rats was conducted to evaluate the intestinal barrier properties of spilanthol in vivo. Rats are one of the most common animals used in preclinical oral absorption studies [36] and are generally considered as good animal models towards humans (*e.g.* F1 modifying factor in permitted daily exposure (PDE) toxicity evaluation is 5 for extrapolation from rats to humans) [38]. Several studies have already shown that there is a good correlation between the absorption of compounds in rat and the absorption in humans [36, 37, 39]*.* Spilanthol was orally administered to rats in a liquid dose solution containing the same solvents as used for DS2 of the Caco-2 cell permeability experiment, consisting of ethanol, PG, vitamin E-TPGS and PEG400. For the absorption rate, the formulation plays an important role and compounds solubilized in liquid generally absorb faster compared to solid forms. The elimination phase, on the contrary, is independent of the oral dosage form. Vitamin E-TPGS, an esterified vitamin E derivative, was added to the formulation because of its solubilizing and emulsifying properties to enhance the solubility and facilitate the absorption of spilanthol [40, 41]. In this in vivo study, spilanthol was clearly observed in the rat serum and hence, spilanthol was able to diffuse through the gut barrier into the systemic circulation, confirming the in vitro Caco-2 cell results. Spilanthol showed an elimination half-life of 1.13 h (63 min), corresponding to similar values reported by Woelkart et al. [17], who investigated the plasma concentration of dodeca-2E,4E,8E,10E/Z-tetraenoic acid isobutylamides from *Echinacea* via oral gavage in rats, resulting in an elimination half-life of 72 min.

Besides the lipophilicity of the compound, its passive gut-blood permeability is dependent on the charge and molecular size of the compound. Considering the lipophilic LogP value of 3.39 of spilanthol and the molecular weight of 221.34 g/mol, it seems that transcellular transport is appropriate for spilanthol [42]. These findings were confirmed in the Caco-2 cell permeability experiment, which indicated that spilanthol diffused through the cells via passive diffusion. Once in the systemic circulation, the investigated compound can distribute to extravascular compartments such as the brain to exert CNS activity.

Therefore, the present study investigated the blood-brain barrier transport properties of spilanthol in an in vivo mice experiment. In kinetic BBB research, one of the ‘golden’ methodologies is the MTR using mice for reasons of costs, handling and animal care. In PDE toxicity evaluation, the F1 modifying factor is 12 for extrapolation from mice to humans [38]. Spilanthol was administered to the mice in a solution containing ethanol, dimethylacetamide and tween 80 in lactated Ringer’s solution containing BSA. Dimethylacetamide and ethanol are often used in injectable pharmaceutical formulations as co-solvents to solubilize poorly soluble compounds, while tween 80 is a traditional surfactant [43, 44]. It is known that tween 80 in high doses up to 30 mg/kg administered to mice causes blood-brain barrier disruption [45]. In this MTR study, a dose of 3 mg/kg tween 80 was used. Also ethanol, at a concentration of 1-4 g/kg, may cause disruption of the BBB as well [45]. However, in the current study, only 0.04 g ethanol/kg mice was administered. Although transient effects cannot be excluded, our data indicate that the solvents did not affect the BBB integrity. In addition, no toxicity was observed during the experiment. From our results, it is concluded that spilanthol was able to rapidly penetrate the BBB after intravenous administration with a unidirectional influx rate of 796.2 μl/(g · min). This high K_1_ value indicates a high initial influx rate of spilanthol into the brains, which is supported by the lipophilic character of this NAA. Moreover, 98 % of spilanthol was found in the parenchyma of the brains, while only 2 % was trapped in the capillaries, which makes it possible for NAAs to exert CNS effects. Furthermore, significant efflux out of the brain into the blood was also observed.

It was already mentioned in the introduction that *Spilanthes acmella* extracts showed some central analgesic activities. In general, different central nervous pharmacodynamic activities are attributed to *N*-alkylamide containing plants. Psychotropic effects (anxiolytic effect) in animals were already found by using alkylamide *Echinacea* preparations, which was explained by their affinity for the CB receptors. Furthermore, similar to cannabinoids, alkylamides alter immune cell activity, which open perspectives for the treatment of neuroinflammatory diseases [17, 46]. In addition, capsaicin, the major NAA in hot peppers of the plant genus Capsicum interacts agonistically with the transient receptor potential vanilloid 1 receptor, predominantly expressed in primary afferent fibres and sensory neurones, playing a role in pain sensation [47, 48]. Another study reported an improvement of amyloid beta(1–42)-induced spatial memory impairment after administration of a 50 mg/kg and 100 mg/kg methanolic extract of *Piper nigrum* fruits by gastric gavage to rats. The plant extract, containing piperine, which is a major alkylamide of black pepper, caused an attenuation of the oxidative stress in the rat hippocampus. Other studies demonstrated analgesic, anticonvulsant, antidepressant, anti-oxidant, anti-inflammatory, protection against neurodegeneration and cognitive enhancing effects of cognitive deficit-like condition of Alzheimer’s disease of piperine [49, 50].

In addition, in some studies there is an indirect proof of blood-brain barrier influx of *N*-alkylamides. It has been shown that several *N*-alkylamide containing plants showed activity against epilepsy in animal models. Experiments with medicinal plants such as *Anacyclus pyrethrum*, *Nigella sativa*, *Ferula gummosa* and *Pimpinalla anisum* were performed to investigate their effect on seizures of epilepsy. It has been shown that the chloroform fraction of *Anacyclus pyrethrum* roots, 100–800 mg/kg administered intraperitoneally to mice, possess neuropharmacological effects, such as antiseizure activity [51, 52]. Other studies demonstrated the myorelaxation and anticonvulsant activities in mice of the *Anacyclus pyrethrum* extract as well [51, 53]. Treatment with capsaicin resulted in a decrease in behavioral seizure activity and body temperature in a kainic acid induced epileptogenesis mice model. Furthermore, anti-oxidant activity in blood increased and the concentrations of IL-1β and TNF-α in the brain lowered [48].

The majority of the studies reported in literature only look at the effects of in vivo or in vitro studies and no exposure data are available. Therefore, more detailed studies about exposure levels are required. In the current study, spilanthol concentrations in brains and serum were quantitatively measured and we demonstrated a high influx of spilanthol into the brain, indicating a possible role in CNS diseases.

## Conclusion

In this study, it is demonstrated that spilanthol is able to cross the Caco-2 cell monolayer in vitro and the intestinal membrane in vivo in rats after oral administration. After absorption and reaching the systemic circulation, spilanthol is able to rapidly and significantly cross the BBB in mice. Spilanthol also showed a significant efflux out of the brain, which explains partly the biphasic behavior of the influx. These results suggest possible medicinal applications for spilanthol in central nervous system diseases from a pharmacokinetic point of view.

## Abbreviations

Ab, apical-to-basolateral; ACN, acetonitrile; Ba, basolateral-to-apical; BBB, blood-brain barrier; BSA, bovine serum albumin; CaCl_2_, Calcium dichloride; CaCl_2_.2H_2_O, calcium chloride dehydrate; CNS, central nervous system; CV, coefficient of variation; DMEM, Dulbecco’s modified Eagle’s medium; DMSO, dimethylsulfoxide; DS1, dose solution 1; DS2, dose solution 2; ESI, electrospray ionisation; θ: exposure time; FA, formic acid; FDC, Franz diffusion cell; GABA, gamma-aminobutyric acid; HBSS, Hanks’ Balanced Salt Solution; HCl, hydrochloric acid; H_3_PO_4_, Phosphoric acid; ICR-CD-1, Institute for Cancer Research Caesarean Derived-1; ICV, intracerebroventriculary; IS, internal standard; K, net clearance; K_1_, unidirectional clearance; k_e_, elimination rate constant; k_out_, efflux rate constant; K_p,aq_, permeability coefficient; KCl, potassium chloride; LLoQ, lower limit of quantification; LoD, limit of detection; LoQ, limit of quantification; MeOH, methanol; MS, mass spectrometer; MRM, multiple reaction monitoring; MTR, multiple time regression; NAAs, *N*-alkylamides; NaCl, sodium chloride; NaHCO_3_, sodium hydrogen carbonate; NaH_2_PO_4_, sodium dihydrogen phosphate; Na_2_HPO_4_.2H_2_O, disodium hydrogen phosphate dehydrate; NaH_2_PO_4_.H_2_O, sodium dihydrogen phosphate monohydrate; NaOH, sodium hydroxide; Na_2_SO_4_, sodium sulphate; P_app_, apparent permeability coefficient; PBS, phosphate buffered saline; PDE, permitted daily exposure; PEG 400, polyethylene glycol 400; PG, propylene glycol; SSR, sum of squared residuals; t_1/2,brain_, efflux brain half-life; TEER, transepithelial electrical resistance; V_0_, vascular brain distribution volume; V_g_, tissue brain distribution volume; H_2_O, water.

## Additional file

Additional file 1: The NMR spectra of isobutyldecanamide. (PDF 284 kb)
