# Supplementary figures and images for: Mucosal and blood-brain barrier transport kinetics of the plant N-alkylamide spilanthol using in vitro and in vivo models
Source: BMC Complement Altern Med. 2016 Jun 13;16:177. doi: 10.1186/s12906-016-1159-0 (PMC4907212; doi:10.1186/s12906-016-1159-0)

***N*-Isobutyldecanamide  $^1\text{H}$  NMR (300 MHz  $\text{CDCl}_3$ )**

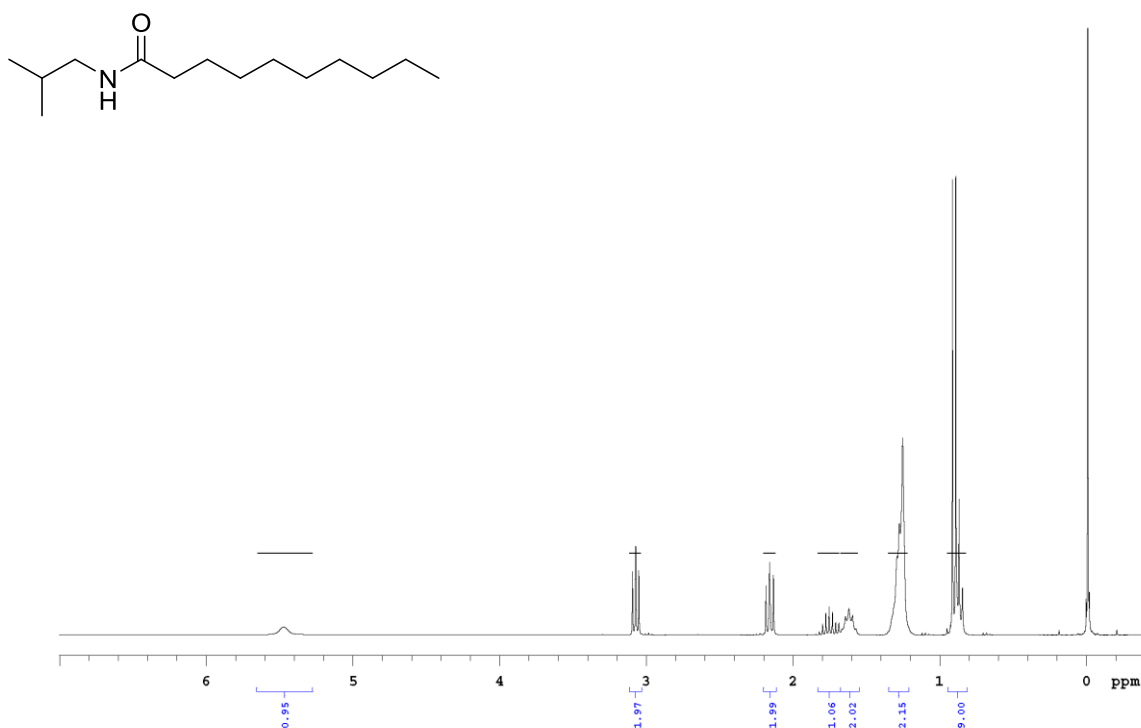

***N*-Isobutyldecanamide  $^{13}\text{C}$  NMR APT (75 MHz  $\text{CDCl}_3$ )**

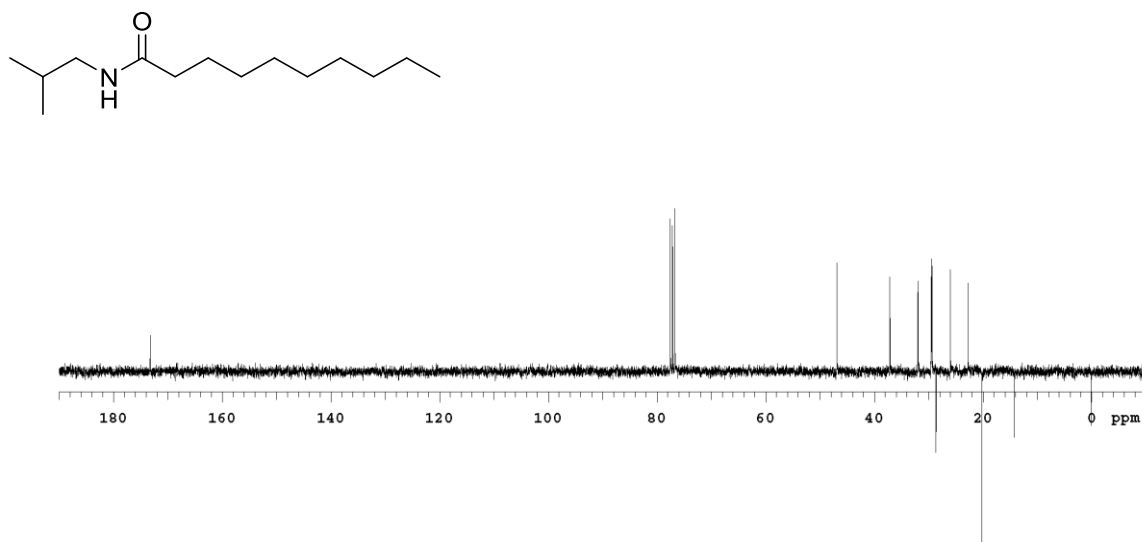

Supplement: Additional file 1: — The NMR spectra of isobutyldecanamide. (PDF 284 kb) [file 12906_2016_1159_MOESM1_ESM.pdf]
